# Metabolic Rate Limits the Effect of Sperm Competition on Mammalian Spermatogenesis

**DOI:** 10.1371/journal.pone.0076510

**Published:** 2013-09-19

**Authors:** Javier delBarco-Trillo, Maximiliano Tourmente, Eduardo R. S. Roldan

**Affiliations:** Reproductive Ecology and Biology Group, Museo Nacional de Ciencias Naturales, Consejo Superior de Investigaciones Científicas, Madrid, Spain; University of Nevada School of Medicine, United States of America

## Abstract

Sperm competition leads to increased sperm production in many taxa. This response may result from increases in testes size, changes in testicular architecture or changes in the kinetics of spermatogenesis, but the impact of each one of these processes on sperm production has not been studied in an integrated manner. Furthermore, such response may be limited in species with low mass-specific metabolic rate (MSMR), i.e., large-bodied species, because they cannot process energy and resources efficiently enough both at the organismic and cellular levels. Here we compare 99 mammalian species and show that higher levels of sperm competition correlated with a) higher proportions of seminiferous tubules, b) shorter seminiferous epithelium cycle lengths (SECL) which reduce the time required to produce sperm, and c) higher efficiencies of Sertoli cells (involved in sperm maturation). These responses to sperm competition, in turn, result in higher daily sperm production, more sperm stored in the epididymides, and more sperm in the ejaculate. However, the two processes that require processing resources at faster rates (SECL and efficiency of Sertoli cells) only respond to sperm competition in species with high MSMR. Thus, increases in sperm production with intense sperm competition occur via a complex network of mechanisms, but some are constrained by MSMR.

## Introduction

Sperm competition takes place when the sperm of two or more males compete to fertilize the ova of a female [1-3]. Due to the prevalence of female promiscuity [4], sperm competition is a pervasive evolutionary force across taxa. In species that experience high levels of sperm competition, males increase their sperm production [5], which allows them to ejaculate more sperm in competitive contexts [6]. Given the direct effect that sperm competition has on male fitness, a general prediction is that many morphological and physiological male traits will evolve in response to the levels of sperm competition, especially those traits that control sperm production [1]. The most straightforward way to increase sperm production is by augmenting the size of the testes [7-9], but males could also maximize their rate of sperm production by adjusting either the architecture of the testis or the kinetics of sperm formation [10,11].

Sperm are produced in the testes through the process of spermatogenesis that takes place within the seminiferous tubules [12], which are separated by interstitial tissue [13]. The seminiferous tubules consist of a seminiferous epithelium, which lines the wall of the tubule, and the lumen, to which mature sperm cells are released. The seminiferous epithelium contains germ cells and their supporting and nourishing cells, the Sertoli cells [13,14].

Several architectural features of the testis could influence sperm production and thus may be affected by sperm competition. On the one hand, an increase in the proportion of testis occupied by seminiferous tubules should increase the rate of sperm production per gram of testis [10,15-17]. On the other hand, three architectural traits of the seminiferous tubules could also impact on sperm production. First, a reduction in tubule diameter could increase the number of tubules per gram of testis, so we hypothesized that high levels of sperm competition may favour a decrease in tubule diameter. Second, a reduction in height of the seminiferous epithelium could result in faster sperm production, so we also hypothesized that high levels of sperm competition may favour a decrease in the height of the seminiferous epithelium. Third, Sertoli cells occupy a large proportion of the seminiferous epithelium, which necessarily reduces the space occupied by germ cells; we hypothesized that high levels of sperm competition could lead to a reduction in the number of Sertoli cells, which would lead to a relative increase in the proportion of the seminiferous epithelium occupied by germ cells (assuming that the size of the remaining Sertoli cells does not increase) and thus to a possible increase in sperm production.

The kinetics of spermatogenesis is species-specific and determined by the seminiferous epithelium cycle length (SECL). The SECL is the time period between two successive occurrences of the same seminiferous stage [18,19]. A shorter SECL leads to a shorter duration of spermatogenesis, and thus to higher rates of sperm production [18]. Spermiogenesis is the last phase of spermatogenesis in which spermatids differentiate into spermatozoa [12]. A decrease in the duration of spermiogenesis should also lead to an increase in sperm production. Sperm production could also increase by elevating the efficiency of Sertoli cells [14,20], defined as the number of round spermatids (the haploid but still immature germ cells) nurtured by each Sertoli cell [14,21]. Consequently, higher levels of sperm competition might lead to a reduction in SECL and spermiogenesis and an increase in the efficiency of Sertoli cells [11,14,22].

Ultimately, the increase in sperm numbers in sperm reserves (caudae epididymides in mammals) and in the ejaculate with high levels of sperm competition [5,15,23] can be due to a combination of responses, including increases in testis size, changes in testis architecture and modifications in the kinetics of spermatogenesis. Importantly, some of these responses may also be affected by mass-specific metabolic rate (MSMR), calculated as the ratio between basal metabolic rate and body mass. For example, in a study comparing six shrew species, a negative relationship was found between MSMR and SECL [22]. Therefore, MSMR can limit the capacity of sperm competition to affect reproductive traits that require a rapid processing of energy and resources at the cellular level [24,25]. This “metabolic rate constraint hypothesis” has received recent support in mammals: in species with high MSMR (e.g., rodents), high levels of sperm competition result in an advantageous increase in sperm size, whereas species with low MSMR (large-bodied mammals) exhibit a metabolic constraint on the evolution of sperm size with high levels of sperm competition [24,25].

Here we analyse for the first time a comprehensive series of traits potentially influencing sperm production, including aspects of testis architecture and kinetics of spermatogenesis (percentage of seminiferous tubules, tubule diameter, height of seminiferous epithelium, number of Sertoli cells, efficiency of Sertoli cells, SECL and spermiogenesis), to assess which may be affected by sperm competition and which by MSMR. We also examined whether traits that require a fast turnover of resources may be differentially affected by sperm competition in species with higher or lower MSMR especially because of metabolic rate constraints.

## Methods

The study sample includes all terrestrial eutherian mammals for which information on the traits related to spermatogenesis were available (*n* = 99). For each trait of interest we made literature searches in Web of Knowledge and Google Scholar. We only used results from studies in which males were adult and reproductively normal. In experimental studies, we only used data from the control groups. The methods used to measure some traits may differ among studies (e.g., most studies used tritiated thymidine to measure SECL, whereas other studies have used bromodeoxyuridine (BrdU), with no differences between methodological approaches, however, being apparent [26]). We did not include two species for the following reasons: a bat, *Pteropus* *poliocephalus* , due to the unusual reproductive traits of Chiroptera which include sperm storage for long periods of time in the female tract, and possible effects of flight on the metabolic rate of this species; and *Dasypus* *novemcinctus* , because its presence introduced an extreme asymmetry at the base of the phylogeny (one branch leading to *Dasypus* and the other branch leading to the remaining 99 species) which could lead to spurious results in phylogenetic analyses [27].

The variables that we analysed in this study and their corresponding sample sizes were the following (see Dataset S1): body mass (g; *n* = 99), testes mass (g; *n* = 99), percentage of the testis occupied by seminiferous tubules (*n* = 62), diameter of the seminiferous tubules (µm; *n* = 66), height of the seminiferous epithelium (µm; *n* = 32), relative number of Sertoli cells (10^6^ Sertoli cells / g testis; *n* = 35), efficiency of Sertoli cells (number of round spermatids / Sertoli cell; *n* = 32), seminiferous epithelium cycle length (SECL; days; *n* = 62), duration of spermiogenesis (days; *n* = 25), daily sperm production (10^6^ sperm/g testis x day; *n* = 36), number of sperm in the cauda epididymides (10^6^ sperm; *n* = 43), number of sperm in the ejaculate (10^6^ sperm; *n* = 44), mass-specific metabolic rate (ml O_2_/h x g; *n* = 67). When more than one value was reported, we calculated an average value weighted by sample size. It must be noted that daily sperm production is normally calculated using a SECL value, and thus these two variables can be partly autocorrelated. All variables were log_10_-transformed to meet parametric assumptions, except for the proportion of testicular tissue occupied by seminiferous tubules, which was arcsine-transformed.

We tested the influence of sperm competition on the architectural and kinetic variables mentioned above using multiple regression analyses in which each variable of interest was a dependent variable and body mass and testes mass were the predictors (this is a more appropriate approach to determine the effect of relative testes mass on a dependent variable than using a "relative testes mass" measure, sensu Kenagy and Trombulak 1986 [28], as the only predictor). We also tested the influence of metabolic rate on those same variables, using mass-specific metabolic rate as predictor. For all these analyses we conducted phylogenetic generalised least squares (PGLS) [29] models in R 2.13.0 [30]. The PGLS estimates a phylogenetic scaling parameter lambda (λ), which is then incorporated in the models to control for phylogenetic effects. If λ values are close to 0, the traits are likely to have evolved independently of phylogeny, whereas values close to 1 indicate strong phylogenetic association of the investigated traits. The phylogenetic reconstruction used in the PGLS analyses is included as Supporting Information (see Figure S1).

We also performed six analyses in which for three dependent variables (SECL, efficiency of Sertoli cells and sperm in the cauda epididymides) we analysed separately species with high and low MSMR. We ordered all species in each dataset by MSMR and selected half of the species with the lowest MSMR values for one analysis and half of the species with the highest MSMR values for another analysis. If the number of species in a dataset was odd, the one species in the middle of the dataset was considered as a low MSMR species if its value was closer to the highest value of the low MSMR species than to the lowest value of the high MSMR species, or as a high MSMR species if its value was closer to the lowest value of the high MSMR species than to the highest value of the low MSMR species. We used this approach to maintain similar sample sizes between the analyses of low MSMR and high MSMR species. The representation of species for each one of the six analyses we performed is the following (for clarity, we use these abbreviations: Art = Artiodactyla; Car = Carnivora; Eul = Eulipotyphla; Lag = Lagomorpha; Per = Perissodactyla; Pri = Primates; Rod = Rodentia): Low MSMR and efficiency of Sertoli cells: 4 Art, 4 Car, 2 Per, 1 Pri, 2 Rod; Low MSMR and SECL: 5 Art, 7 Car, 1 Eul, 2 Per, 5 Pri, 3 Rod; Low MSMR and sperm in cauda: 4 Art, 1 Car, 1 Lag, 2 Per, 2 Pri, 6 Rod; High MSMR and efficiency of Sertoli cells: 2 Car, 1 Lag, 3 Pri, 6 Rod; High MSMR and SECL: 1 Car, 6 Eul, 1 Lag, 3 Pri, 13 Rod; High MSMR and sperm in cauda: 6 Eul, 9 Rod.

**Figure 1:**
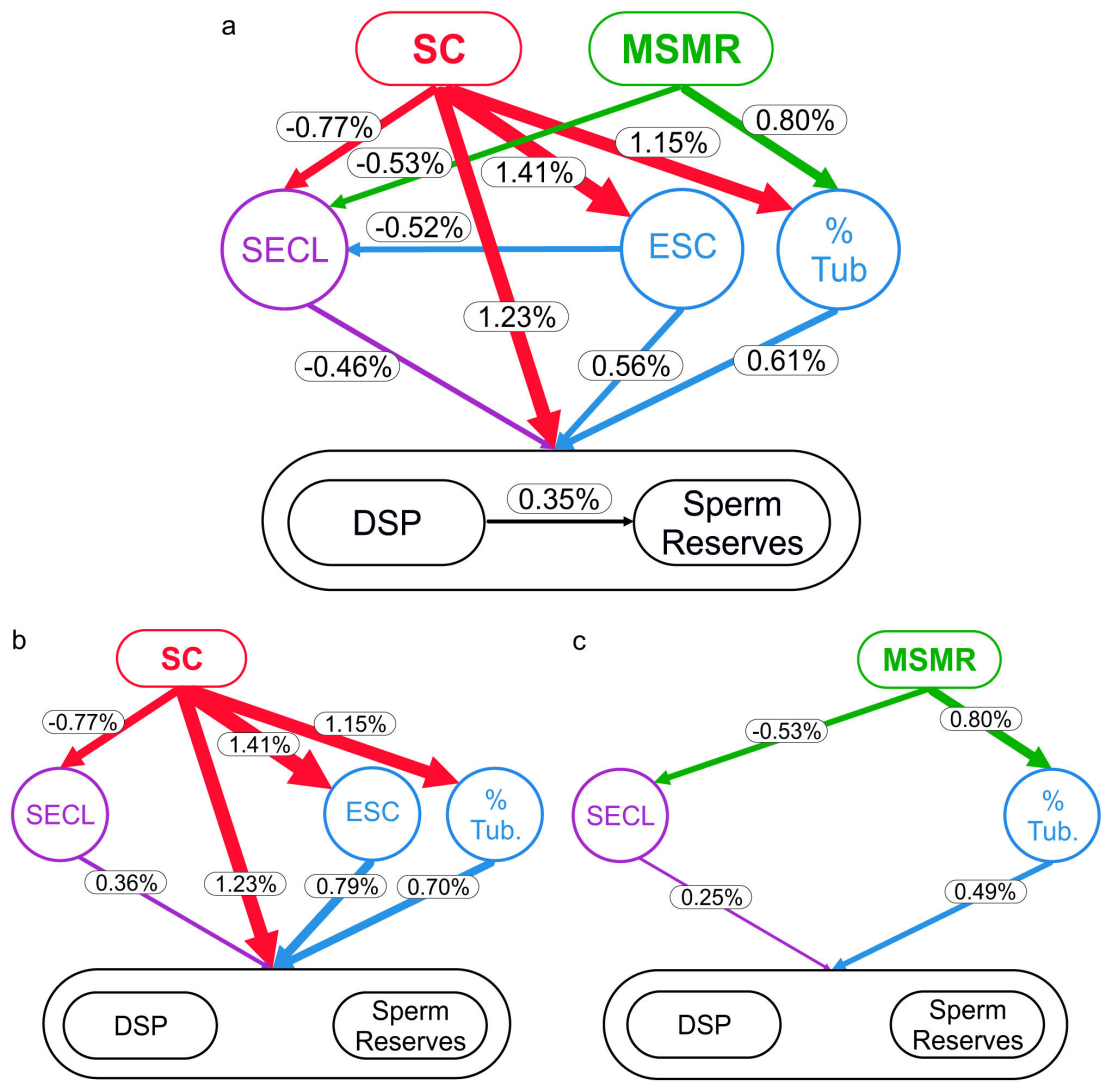
Schematic representation of the relationships between sperm competition, mass-specific metabolic rate, testicular architecture, kinetics of spermatogenesis, sperm production and numbers in sperm reserves in eutherian mammals. **a**, Static 1% variations: numbers next to arrows are the relative variation in the dependent variable caused by a variation of 1% of the sample range in the independent variable. For example, there is a -0.77% decrease in SECL when we increase SC by 1%. **b**, **c**, Dynamic variations: numbers next to arrows between level 1 and level 2 variables are the relative variation in the dependent variable caused by a variation of 1% of the sample range in the independent variable (thus these values are the same as in panel a); numbers next to arrows between level 2 and level 3 variables are the relative variation in the dependent variable (level 3) caused by the change in the independent variable (level 2) due to a 1% increment in level 1. All relative variations are presented as percentages of the sample range. Relative variation percentages were calculated using the slopes and intercepts estimated by PGLS models. Arrow widths are proportional to indicated magnitudes. Abbreviations: SC: sperm competition (relative testes size); MSMR: mass-specific metabolic rate; SECL: seminiferous epithelium cycle length; ESC: efficiency of Sertoli cells; % Tub: percentage of the testicular tissue occupied by seminiferous tubules; DSP: daily sperm production; Sperm Reserves: number of spermatozoa in the caudae epididymides.

In an effort to clarify the relative contribution of the variations in testis architecture and kinetics of spermatogenesis (in response to sperm competition or MSMR) to sperm production, we calculated the impact that theoretical variations of the predictor variables would have on the values of the dependent variables. Thus, we identified three hierarchic levels of variables based on the hypothetic relationships tested in the models (see Figure 1): 1) sperm competition and MSMR; 2) SECL, efficiency of Sertoli cells, and percentage of the testis occupied by seminiferous tubules; and 3) daily sperm production, and number of sperm in the cauda epididymides. We then used the slopes and intercepts estimated by the PGLS models to predict the relative influence of one level on the successive levels. We applied two different approaches. First, we used static variations: we introduced an increase in a predictor variable at level 1 equivalent to 1% of its range (i.e., minimum to maximum), and the resulting variation on each level-2 dependent variable was calculated (also as a percentage of that variable’s range). For example, in Figure 1a it can be seen that an increase in MSMR equivalent to 1% of MSMR’s range would produce a decrease in SECL equivalent to 0.53% of SECL’s range. We performed the same type of calculations using the relationships between level 2 and level 3 variables: we introduced an increase in a predictor variable at level 2 equivalent to 1% of its range, and the resulting variation on the level 3 dependent variable was calculated. Second, we used dynamic variations: the calculation procedure was similar to the one used in static variations. However, for the relationships between level 2 and 3, the variation (% of range) introduced in the predictor variable at level 2 was the result of a variation of 1% in the range in the level 1 predictor. For example, in Figure 1c it can be seen that an increase in MSMR equivalent to 1% of MSMR’s range would produce a decrease in SECL equivalent to 0.53% of SECL’s range, and that such a 0.53% decrease in SECL would produce a 0.25% increase in the range of sperm numbers in cauda. All variations are expressed as a percentage of the sample range.

## Results

There was no significant relationship between MSMR and relative testes size among species in this study (phylogenetic generalised least squares, PGLS: *P*>0.05; Table S1). Therefore, in those cases in which both MSMR and relative testes size have significant effects on variables related to the output of spermatogenesis, we consider these effects to be mostly independent from each other.

We found that the percentage of testicular tissue comprising seminiferous tubules increased with relative testes size (PGLS: *P*=0.0002) and with MSMR (PGLS: *P*=0.0001) (Table 1). However, neither relative testes size nor MSMR had any effect on the diameter of seminiferous tubules, the height of the seminiferous epithelium, or the number of Sertoli cells per gram of testis (PGLS: *P*>0.05; Table 1).

**Table 1:** Effects of sperm competition and metabolic rate on spermatogenic traits.

Dependent variable	Predictor	Slope	*F*	*P* value	*λ*	*r*	CI	n
% of seminiferous tubules	RTS^a^	0.19	16.04	**0.0002**	0.79^*, n.s.^	0.46	**0.25 to 0.76**	62
	MSMR	0.37	18.54	**0.0001**	0.25^n.s., *^	0.59	**0.34 to 1.01**	37
Tubule diameter	RTS^a^	0.03	1.45	0.23	0.71^*, n.s.^	0.15	-0.10 to 0.40	66
	MSMR	0.04	0.96	0.33	0.24^n.s., *^	0.15	-0.16 to 0.46	43
Height of epithelium	RTS^a^	0.05	1.05	0.31	0.50^n.s., n.s.^	0.19	-0.17 to 0.55	32
	MSMR	-0.08	3.80	0.067	<0.01^n.s., n.s.^	0.42	-0.03 to 0.92	20
Number of Sertoli cells	RTS^a^	-0.18	0.11	0.12	0.67^n.s., n.s.^	0.06	-0.29 to 0.41	35
	MSMR	-0.14	0.91	0.35	0.23^n.s., *^	0.19	-0.21 to 0.59	27
Efficiency of Sertoli cells	RTS^a^	0.29	15.98	**0.0004**	<0.01^n.s., *^	0.60	**0.32 to 1.05**	32
	MSMR	0.13	1.30	0.27	<0.01^n.s., *^	0.23	-0.18 to 0.65	25
SECL	RTS^a^	-0.07	5.48	**0.02**	<0.01^n.s., *^	0.29	**0.05 to 0.56**	62
	MSMR	-0.10	7.59	**0.008**	0.76^*, n.s.^	0.38	**0.10 to 0.70**	47
Spermiogenesis	RTS^a^	-0.03	0.34	0.56	<0.01^n.s., *^	0.12	-0.29 to 0.54	25
	MSMR	-0.06	1.31	0.27	<0.01^n.s., *^	0.26	-0.21 to 0.74	20
Daily sperm production	RTS^a^	0.26	8.58	**0.006**	0.999^*, n.s.^	0.45	**0.15 to 0.83**	36
	MSMR	0.21	1.85	0.19	<0.01^n.s., *^	0.27	-0.14 to 0.70	25
Sperm in cauda	RTS^a^	1.70	116.31	**<0.0001**	0.59^n.s., *^	0.86	**0.99 to 1.61**	43
	MSMR	-0.99	2.94	0.097	0.999^*, n.s.^	0.30	-0.06 to 0.68	31
Sperm in ejaculate	RTS^a^	1.58	32.21	**<0.0001**	<0.01^n.s., *^	0.66	**0.49 to 1.11**	44
	MSMR	-1.22	3.48	0.071	0.999^n.s., n.s.^	0.30	-0.03 to 0.65	37

Phylogenetically controlled multiple regression analyses revealing the effects of relative testes mass (RTS) and mass-specific metabolic rate (MSMR) on spermatogenic traits. ^a^ In the RTS analyses, we report the values for the second predictor (testes mass) after controlling for the effect of the first predictor (body mass; see Table S2 for the values of body mass). All variables were log_10_ transformed (with the exception of the proportion of seminiferous tubules, which was arcsine transformed) prior to analysis. The superscripts following the λ value indicate significance levels (n.s., p > 0.05; * p < 0.05) in likelihood ratio tests against models with λ = 0 (first superscript) and λ = 1 (second superscript). The effect size r was calculated from the F values; we also present the non-central 95% confidence interval (CI), an interval excluding 0 indicating statistically significant relationships. The P values and CI that indicate statistical significance are shown in bold. Abbreviations: n: number of species in each analysis; SECL: seminiferous epithelium cycle length.

We also found that SECL, and thus the duration of spermatogenesis, decreased as relative testes size increased (PGLS: *P*=0.023; Figure 1, Table 1) and when MSMR also increased (PGLS: *P*=0.008). Neither relative testes size nor MSMR were associated with the duration of spermiogenesis (PGLS: *P*>0.05), despite the strong relationship between SECL and the duration of spermiogenesis (PGLS: *P*<0.0001, R^2^ adjusted=0.9).

The efficiency of Sertoli cells increased in species with higher relative testes size (PGLS: *P*=0.0004), but not in relation to MSMR (PGLS: *P*=0.27). A higher efficiency of the Sertoli cells was associated with shorter SECL (PGLS: *P*=0.014) and shorter duration of spermiogenesis (PGLS: *P*=0.01; Table S3).

All the spermatogenic traits affected by high levels of sperm competition had, in turn, positive effects on sperm production after removing the effect of body size (Figure 1). That is, the increased percentage of seminiferous tubules, the reduced SECL, and the increased efficiency of Sertoli cells in response to high levels of sperm competition, were all associated with a higher daily sperm production per gram of testis (PGLS: *P*<0.0005 for all analyses), and a higher number of sperm stored in the caudae epididymides (PGLS: *P*<0.005 for all analyses; Table S4).

Furthermore, when using phylogenetically-corrected linear model equations to predict variation in sperm production variables, we realised that there are important differences in the relative degree in which the different traits underlying testis architecture and kinetics of spermatogenesis influence total sperm production (Figure 1). Sperm production and sperm reserves were similarly affected by a static 1% increase in SECL (-0.46%), in Sertoli cells efficiency (0.56%) and in the percentage of tubules (0.61%; Figure 1a). However, a variation of 1% in relative testes mass produced more than 1% variation in seminiferous tubules (1.15%) and Sertoli cells efficiency (1.41%), but less than 1% variation in SECL (0.77%; Figure 1a). Consequently, the relative dynamic variation in daily sperm production and sperm reserves caused by the effect of sperm competition on testicular architecture variables (0.79% for Sertoli cells efficiency; 0.70% for tubule percentage) would at least double that caused by the effect of sperm competition on kinetics of spermatogenesis, i.e., SECL (0.36%; Figure 1b). A similar pattern was detected when analysing the effect of MSMR as the cause of variation in the percentage of tubules (architecture) and SECL (kinetics) (Figure 1c).

Species with higher daily sperm production have more sperm stored in the caudae epididymides (PGLS: *P*=0.039), and, in turn, more sperm stored in the caudae leads to a higher number of sperm in the ejaculate (PGLS: *P*<0.0001; Table S4). On the other hand, MSMR was not directly associated with daily sperm production, sperm in the caudae, or sperm in the ejaculate (PGLS: *P*>0.05).

Finally, we analysed separately species with low and high MSMR to assess how species may adjust their spermatogenic traits in response to sperm competition. In species with high MSMR, increased sperm competition was associated with shorter SECL (PGLS: *P*=0.002) and a higher efficiency of the Sertoli cells (PGLS: *P*=0.005). This was not the case for species with low MSMR (PGLS: *P*>0.05 for all analyses; Table 2). Higher levels of sperm competition ultimately led to greater numbers of sperm in the caudae regardless of whether species had low MSMR (PGLS: *P*=0.0002) or high MSMR (PGLS: *P*<0.0001).

**Table 2:** Effects of sperm competition on spermatogenic traits depending on metabolic rate.

MSMR	Dependent variable	Predictor	Slope	*F*	*P* value	*λ*	*r*	CI	n
Low	ESC	body mass	-0.17	0.20	0.66	<0.01^n.s., n.s.^	0.14	-0.48 to 0.76	13
		testes mass	0.27	2.86	0.12		0.47	-0.11 to 1.13	
Low	SECL	body mass	0.07	4.25	0.053	<0.01^n.s., n.s.^	0.42	**0.01 to 0.88**	23
		testes mass	-0.06	2.10	0.16		0.31	-0.12 to 0.76	
Low	SpCauda	body mass	-0.61	0.41	0.53	0.84^n.s., n.s.^	0.18	-0.37 to 0.72	16
		testes mass	1.61	26.29	**0.0002**		0.82	**0.61 to 1.69**	
High	ESC	body mass	-0.29	7.53	**0.023**	<0.01^n.s., *^	0.68	**0.17 to 1.47**	12
		testes mass	0.37	14.02	**0.005**		0.78	**0.39 to 1.70**	
High	SECL	body mass	0.15	7.00	**0.015**	0.94^*, n.s.^	0.50	**0.12 to 0.98**	24
		testes mass	-0.13	12.19	**0.002**		0.61	**0.28 to 1.13**	
High	SpCauda	body mass	-0.54	36.52	**<0.0001**	<0.01^n.s., n.s.^	0.87	**0.76 to 1.89**	15
		testes mass	1.67	39.7	**<0.0001**		0.88	**0.79 to 1.93**	

Phylogenetically controlled multiple regression analyses revealing the effect of relative testes mass on spermatogenic traits in species with low and high mass-specific metabolic rates (MSMR). All variables were log_10_-transformed prior to analysis. The superscripts following the λ value indicate significance levels (n.s., p > 0.05; * p < 0.05) in likelihood ratio tests against models with λ = 0 (first superscript) and λ = 1 (second superscript). The effect size *r* was calculated from the *F* values; we also present the non-central 95% confidence interval (CI), an interval excluding 0 indicating statistically significant relationships. The *P* values and CI that indicate statistical significance are shown in bold. Abbreviations: n: number of species in each analysis; ESC: efficiency of Sertoli cells (number of round spermatids / Sertoli cell); SECL: seminiferous epithelium cycle length; SpCauda: number of sperm in the cauda epididymides.

## Discussion

The overall result of our study is that sperm competition leads to an increase in sperm production by affecting several testicular traits. Therefore, high levels of sperm competition promote a higher production of sperm not only by increasing the size of the testes [8,28], but also by affecting morphological and kinetic traits within the testes that have a direct influence on sperm production. These traits are the percentage of testicular tissue occupied by seminiferous tubules, the efficiency of the Sertoli cells, and the seminiferous epithelium cycle length (SECL) and thus the duration of spermatogenesis.

First, we found that high levels of sperm competition are associated with an increase in the percentage of seminiferous tubules in mammals. This expands earlier observations in rodents [Gómez Montoto L, Arregui L, Roldan ERS; unpublished data] and birds [10,15], which strengthens the straightforward argument that an increase in the proportion of sperm-producing tissue will lead to an increase in the numbers of sperm produced.

Second, we also found that another route to increase sperm production in response to sperm competition is to increase the efficiency of Sertoli cells. In fact, increasing the efficiency of the Sertoli cells is as effective in augmenting sperm production as increasing the percentage of seminiferous tubules. An increase in the efficiency of Sertoli cells in relation to high levels of sperm competition has also been shown in birds [14].

Third, another way to increase sperm production in response to sperm competition is decreasing SECL and thus the duration of spermatogenesis [11]. Our data supports this relationship and thus agree with previous studies that compared a small number of species, e.g., six shrew species [22] and two rodent species [23]. We also found that higher efficiencies of the Sertoli cells are associated with a decrease in SECL. It is thus possible that the reduced SECL in response to high levels of sperm competition is due to the concomitant increase in the efficiency of the Sertoli cells.

Our results indicate that the contributions of (a) the proportion of seminiferous tubules, (b) the efficiency of Sertoli cells and (c) SECL to sperm production in response to the same level of sperm competition are not symmetrical. Similar changes in the proportion of seminiferous tubules and the efficiency of Sertoli cells resulted in twice as much increase in sperm production compared to SECL. This may be the case because an increase in both the proportion of seminiferous tubules and the efficiency of Sertoli cells would result in a direct increase in the number of germ cells produced per gram of testis. In contrast, the reduction in SECL may result in only a partial acceleration of spermatogenesis because spermiogenesis (the post-meiotic stage of spermatogenesis) is not significantly affected by sperm competition.

All the spermatogenic traits affected by sperm competition (percentage of seminiferous tubules, efficiency of Sertoli cells and SECL) were associated with a higher daily sperm production per gram of testis. Consequently, a combination of increasing the percentage of sperm-producing tissue and increasing the efficiency and speed of the sperm-producing process (spermatogenesis) offers more potential and flexibility for increasing sperm production when required, for example in contexts with high levels of sperm competition. The importance of the increase in daily sperm production in species with high levels of sperm competition is illustrated by the fact that species with higher daily sperm production had more sperm stored in the caudae epididymides, which, in turn, allows males to allocate a higher number of sperm in the ejaculate, as also suggested in previous studies [5].

It is also important to consider which traits were not associated with different levels of sperm competition. These traits were the diameter of the seminiferous tubules, the height of the seminiferous epithelium, and the number of Sertoli cells per gram of testis. One explanation is that these traits may not be able to evolve readily in response to external factors without impairing the normal process of spermatogenesis.

MSMR and relative testes size were not associated with each other, which suggests that the effects of MSMR on testicular traits may be independent from those due to sperm competition. Our results agree with the overall hypothesis that sperm competition is the ultimate factor affecting the up or down-regulation of sperm production, whereas MSMR would be a proximate factor that would constrain such regulations [22]. It must be noted that even though MSMR is associated with some traits that can affect sperm production, MSMR itself is not directly associated with daily sperm production, sperm in the caudae, or sperm in the ejaculate. Species with low MSMR (i.e., with large body sizes), have a lower proportion of testicular tissue occupied by seminiferous tubules and longer SECL. Large-bodied animals have larger testes, independently of sperm competition levels, simply due to allometric reasons [13]; given that the interstitial tissue contains the blood and lymph vessels, macrophages, and Leydig cells, an increase in the volume of the testes may require, in turn, an increase in the percentage of interstitial tissue to guarantee normal testicular function [12,13]. The negative association between MSMR and SECL is even more straightforward. In species with high MSMR, the metabolism of all cell types involved in spermatogenesis will be increased, which will facilitate a faster activity of the spermatogenic processes and thus shorter SECL and spermatogenesis duration [22].

Finally, we found that increased sperm competition was associated with shorter SECL and a higher efficiency of the Sertoli cells in species with high MSMR, but not in those with low MSMR. Nevertheless, higher levels of sperm competition ultimately led to greater numbers of sperm in the caudae regardless of whether species had high or low MSMR. These results suggest that all species, regardless of their MSMR, have the potential to adjust sperm production in response to sexual selection. However, MSMR determines the pathway that different species take to increase sperm numbers under sexual selection. Species with low MSMR are constrained in their ability to respond to sexual selection when such a response involves fast processing rates of energy and resources [25]; in contrast, species with high MSMR are not subjected to such constraints and have the potential to adjust more traits, e.g., decreasing SECL and increasing the efficiency of Sertoli cells. Overall, our results generalize the “metabolic rate constraint hypothesis” [25] to sperm production and show that MSMR limits how increases in sperm production can be attained under sexual selection.

## Supporting Information

Figure S1 **Phylogenetic reconstruction for the 99 eutherian mammal species utilised in the PGLS analyses.**
(DOC)

Table S1 **Relationship between MSMR and relative testes size.**
(DOC)

Table S2 **Effects of sperm competition on spermatogenic traits.**
(DOC)

Table S3 **Relationships among testicular and spermatogenic traits.**
(DOC)

Table S4 **Effects of spermatogenic traits on indicators of sperm production.**
(DOC)

Dataset S1 **The complete dataset used in all the analyses: morphological, reproductive, and metabolic data.**
(XLS)
