# Supplementary material for: Metabolic Rate Limits the Effect of Sperm Competition on Mammalian Spermatogenesis
Source: PLoS One. 2013 Sep 19;8(9):e76510. doi: 10.1371/journal.pone.0076510 (PMC3777943; doi:10.1371/journal.pone.0076510)
Supplement: Figure S1 — Phylogenetic reconstruction for the 99 eutherian mammal species utilised in the PGLS analyses. (DOC) [file pone.0076510.s001.doc]

**Figure** **S1.** Phylogenetic reconstruction for the 99 eutherian mammal species utilised in the PGLS analyses: Due to the unavailability of a complete phylogeny for all analysed species, we used a recently reconstructed phylogeny (Gomendio et al. 2011), which we complemented with trees for the higher groups (orders and families) of the Eutheria (Bininda-Emonds et al. 2007; Prasad et al. 2008), and with trees for the species relationships in Canidae (Valdespino 2007) and Rodentia (Rowe and Honeycutt 2002; Ford 2006; Álvarez et al. 2011; Gómez Montoto et al. 2011). This reconstructed phylogeny does not include any polytomy.


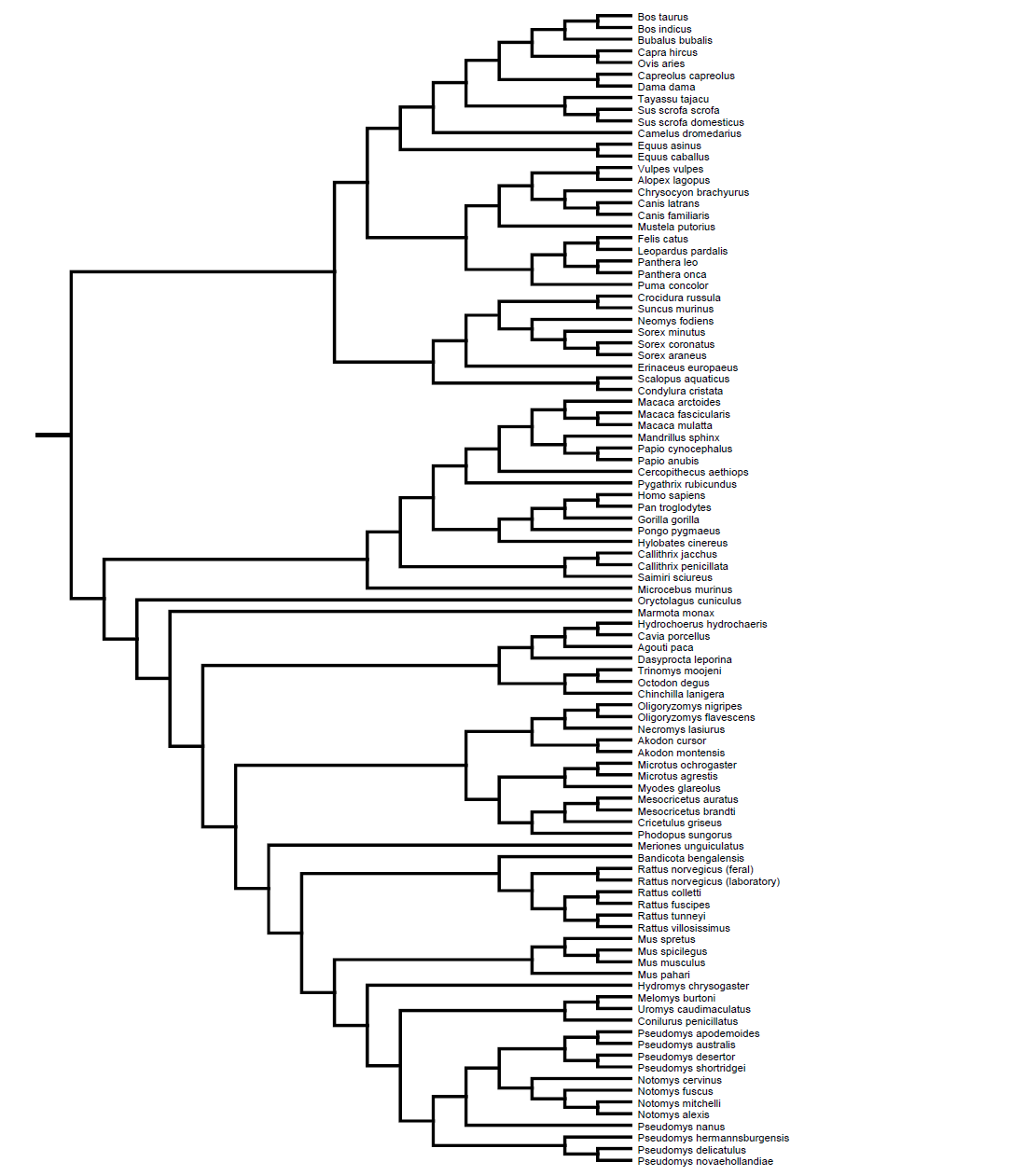


**References**

Álvarez, A., S. I. Perez, and D. H. Verzi. 2011. Ecological and phylogenetic influence on mandible shape variation of South American caviomorph rodents (Rodentia: Hystricomorpha). Biol. J. Linn. Soc. 102:828-837.

Bininda-Emonds, O. R. P., M. Cardillo, K. E. Jones, R. D. E. MacPhee, R. M. D. Beck, R. Grenyer, S. A. Price, R. A. Vos, J. L. Gittleman, and A. Purvis. 2007. The delayed rise of present-day mammals. Nature 446:507-512.

Ford, F. 2006. A splitting headache: relationships and generic boundaries among Australian murids. Biol. J. Linn. Soc. 89:117-138.

Gomendio, M., M. Tourmente, and E. R. S. Roldan. 2011. Why mammalian lineages respond differently to sexual selection: metabolic rate constrains the evolution of sperm size. Proc. R. Soc. Lond. B 278:3135-3141.

Gómez Montoto, L., C. Magaña, M. Tourmente, J. Martín-Coello, C. Crespo, J. J. Luque-Larena, M. Gomendio, and E. R. S. Roldan. 2011. Sperm competition, sperm numbers and sperm quality in muroid rodents. Plos One 6:e18173.

Prasad, A. B., M. W. Allard, N. C. S. Program, and E. D. Green. 2008. Confirming the phylogeny of mammals by use of large comparative sequence data sets. Mol. Biol. Evol. 25:1795-1808.

Rowe, D. L., and R. L. Honeycutt. 2002. Phylogenetic relationships, ecological correlates, and molecular evolution within the Cavioidea (Mammalia, Rodentia). Mol. Biol. Evol. 19:263-277.

Valdespino, C. 2007. Physiological constraints and latitudinal breeding season in the Canidae. Physiol. Biochem. Zool. 80:580-591.
