# Supplementary material for: Metabolic Rate Limits the Effect of Sperm Competition on Mammalian Spermatogenesis
Source: PLoS One. 2013 Sep 19;8(9):e76510. doi: 10.1371/journal.pone.0076510 (PMC3777943; doi:10.1371/journal.pone.0076510)
Supplement: Table S1 — Relationship between MSMR and relative testes size. (DOC) [file pone.0076510.s002.doc]

**Table S1**. Relationship between MSMR and relative testes size

| Dependent variable | Predictor | Slope | *F* | *P* value | *λ* | *r* | CI | n |
| --- | --- | --- | --- | --- | --- | --- | --- | --- |
| MSMR | body mass | -0.29 | 141.98 | **<0.0001** | 1^*, n.s^. | 0.83 | **0.94 to 1.43** | 67 |
|  | testes mass | 0.04 | 0.52 | 0.47 |  | 0.09 | -0.16 to 0.34 |  |

Phylogenetically controlled multiple regression analysis revealing the effect of relative testes mass on mass-specific metabolic rate (MSMR). All variables were log_10_-transformed prior to analysis. The superscripts following the λ value indicate significance levels (n.s., p > 0.05; *, p < 0.05) in likelihood ratio tests against models with *λ* = 0 (first superscript) and *λ* = 1 (second superscript). The effect size *r* was calculated from the *F* values; we also present the non-central 95% confidence interval (CI), an interval excluding 0 indicating statistically significant relationships. The *P* values and CI that indicate statistical significance are shown in bold.
