# Supplementary material for: Metabolic Rate Limits the Effect of Sperm Competition on Mammalian Spermatogenesis
Source: PLoS One. 2013 Sep 19;8(9):e76510. doi: 10.1371/journal.pone.0076510 (PMC3777943; doi:10.1371/journal.pone.0076510)
Supplement: Table S2 — Effects of sperm competition on spermatogenic traits. (DOC) [file pone.0076510.s003.doc]

**Table S2**. Effects of sperm competition on spermatogenic traits

| Dependent variable | Predictor | Slope | *F* | *P* value | *λ* | *r* | CI | n |
| --- | --- | --- | --- | --- | --- | --- | --- | --- |
| % of seminiferous tubules | body mass | -0.25 | 24.01 | **<0.0001** | 0.79^*, n.s^. | 0.54 | **0.35 to 0.86** | 62 |
|  | testes mass | 0.19 | 16.04 | **0.0002** |  | 0.46 | **0.25 to 0.76** |  |
| Tubule diameter | body mass | -0.03 | 1.46 | 0.098 | 0.71^*, n.s^. | 0.15 | -0.10 to 0.40 | 66 |
|  | testes mass | 0.03 | 1.45 | 0.23 |  | 0.15 | -0.10 to 0.40 |  |
| Height of epithelium | body mass | -0.02 | 1.13 | 0.30 | 0.50^n.s., n.s^. | 0.19 | -0.17 to 0.56 | 32 |
|  | testes mass | 0.05 | 1.05 | 0.31 |  | 0.19 | -0.17 to 0.55 |  |
| Number of Sertoli cells | body mass | 0.15 | 0.72 | 0.40 | 0.67^n.s., n.s^. | 0.15 | -0.20 to 0.50 | 35 |
|  | testes mass | -0.18 | 0.11 | 0.12 |  | 0.06 | -0.29 to 0.41 |  |
| Efficiency of Sertoli cells | body mass | -0.23 | 4.09 | 0.052 | <0.01^n.s., *^ | 0.35 | **0.003 to 0.73** | 32 |
|  | testes mass | 0.29 | 15.98 | **0.0004** |  | 0.60 | **0.32 to 1.05** |  |
| SECL | body mass | 0.07 | 9.98 | **0.003** | <0.01^n.s., *^ | 0.38 | **0.15 to 0.66** | 62 |
|  | testes mass | -0.07 | 5.48 | **0.02** |  | 0.29 | **0.05 to 0.56** |  |
| Spermiogenesis | body mass | 0.02 | 0.06 | 0.82 | <0.01^n.s., *^ | 0.05 | -0.37 to 0.47 | 25 |
|  | testes mass | -0.03 | 0.34 | 0.56 |  | 0.12 | -0.29 to 0.54 |  |
| Daily sperm production | body mass | -0.37 | 12.11 | **0.001** | 0.999^*, n.s^. | 0.52 | **0.23 to 0.92** | 36 |
|  | testes mass | 0.26 | 8.58 | **0.006** |  | 0.45 | **0.15 to 0.83** |  |
| Sperm in cauda | body mass | -0.64 | 63.57 | **<0.0001** | 0.59^n.s., *^ | 0.78 | **0.74 to 1.36** | 43 |
|  | testes mass | 1.70 | 116.31 | **<0.0001** |  | 0.86 | **0.99 to 1.61** |  |
| Sperm in ejaculate | body mass | -0.42 | 72.64 | **<0.0001** | <0.01^n.s., *^ | 0.80 | **0.79 to 1.40** | 44 |
|  | testes mass | 1.58 | 32.21 | **<0.0001** |  | 0.66 | **0.49 to 1.11** |  |

Phylogenetically controlled multiple regression analyses revealing the effect of relative testes mass on spermatogenic traits. The data for relative testes mass (testes mass) is also presented in Table 1 (with relative testes mass named RTS). All variables were log_10_-transformed (with the exception of the proportion of seminiferous tubules, which was arcsine-transformed) prior to analysis. The superscripts following the λ value indicate significance levels (n.s., p > 0.05; *, p < 0.05) in likelihood ratio tests against models with *λ* = 0 (first superscript) and *λ* = 1 (second superscript). The effect size *r* was calculated from the *F* values; we also present the non-central 95% confidence interval (CI), an interval excluding 0 indicating statistically significant relationships. The *P* values and CI that indicate statistical significance are shown in bold. Abbreviations: n: number of species in each analysis; SECL: seminiferous epithelium cycle length.
