# Supplementary material for: Metabolic Rate Limits the Effect of Sperm Competition on Mammalian Spermatogenesis
Source: PLoS One. 2013 Sep 19;8(9):e76510. doi: 10.1371/journal.pone.0076510 (PMC3777943; doi:10.1371/journal.pone.0076510)
Supplement: Table S3 — Relationships among testicular and spermatogenic traits. (DOC) [file pone.0076510.s004.doc]

**Table S3**. Relationships among testicular and spermatogenic traits

| Dependent variable | Predictor | Slope | *F* | *P* value | *λ* | *r* | CI | n |
| --- | --- | --- | --- | --- | --- | --- | --- | --- |
| SECL | body mass | -0.01 | 1.29 | 0.26 | 0.80^n.s., n.s^. | 0.19 | -0.14 to 0.53 | 37 |
|  | PST | -0.21 | 2.47 | 0.13 |  | 0.26 | -0.07 to 0.60 |  |
| Spermiogenesis | body mass | -0.02 | 0.06 | 0.81 | <0.01^n.s., *^ | 0.07 | -0.46 to 0.59 | 17 |
|  | PST | -0.20 | 1.20 | 0.29 |  | 0.28 | -0.24 to 0.81 |  |
| SECL | body mass | 0.03 | 6.44 | **0.015** | 0.37^n.s., *^ | 0.37 | **0.08 to 0.70** | 43 |
|  | tubule diameter | 0.29 | 2.53 | 0.12 |  | 0.24 | -0.06 to 0.56 |  |
| Spermiogenesis | body mass | 0.00 | <0.01 | 0.97 | <0.01^n.s., *^ | 0.01 | -0.48 to 0.50 | 19 |
|  | tubule diameter | 0.24 | 0.48 | 0.50 |  | 0.17 | -0.32 to 0.66 |  |
| SECL | body mass | 0.01 | 1.33 | 0.26 | 0.61^n.s., n.s^. | 0.24 | -0.17 to 0.66 | 25 |
|  | HSE | 0.61 | 10.28 | **0.025** |  | 0.56 | **0.22 to 1.06** |  |
| Spermiogenesis | body mass | -0.02 | 0.04 | 0.86 | <0.01^n.s., n.s^. | 0.11 | -1.02 to 1.24 | 6 |
|  | HSE | 1.78 | 17.44 | **0.025** |  | 0.92 | **0.48 to 2.75** |  |
| SECL | number of Sertoli cells | -0.09 | 1.25 | 0.27 | 0.43^n.s., *^ | 0.20 | -0.16 to 0.57 | 32 |
| Spermiogenesis | number of Sertoli cells | 0.06 | 0.34 | 0.57 | <0.01^n.s., *^ | 0.17 | -0.45 to 0.80 | 13 |
| SECL | ESC | -0.21 | 6.92 | **0.014** | 0.17^n.s., *^ | 0.45 | **0.10 to 0.86** | 30 |
| Spermiogenesis | ESC | -0.35 | 9.76 | **0.01** | <0.01^n.s., *^ | 0.70 | **0.22 to 1.53** | 12 |
| Spermiogenesis | body mass | -0.02 | 5.17 | **0.033** | 0.19^n.s., n.s^. | 0.44 | **0.05 to 0.89** | 25 |
|  | SECL | 1.05 | 224.9 | **<0.0001** |  | 0.95 | **1.46 to 2.30** |  |

Phylogenetically controlled multiple regression analyses revealing the effect of testicular traits on SECL and spermiogenesis. All variables were log_10_-transformed (with the exception of the proportion of seminiferous tubules, which was arcsine-transformed) prior to analysis. The superscripts following the λ value indicate significance levels (n.s., p > 0.05; *, p < 0.05) in likelihood ratio tests against models with *λ* = 0 (first superscript) and *λ* = 1 (second superscript). The effect size *r* was calculated from the *F* values; we also present the non-central 95% confidence interval (CI), an interval excluding 0 indicating statistically significant relationships. The *P* values and CI that indicate statistical significance are shown in bold. Abbreviations: SECL: seminiferous epithelium cycle length; n: number of species in each analysis; PST: percentage of seminiferous tubules; HSE: height of the seminiferous epithelium; ESC: efficiency of Sertoli cells (number of round spermatids / Sertoli cell).
