# Supplementary material for: Metabolic Rate Limits the Effect of Sperm Competition on Mammalian Spermatogenesis
Source: PLoS One. 2013 Sep 19;8(9):e76510. doi: 10.1371/journal.pone.0076510 (PMC3777943; doi:10.1371/journal.pone.0076510)
Supplement: Table S4 — Effects of spermatogenic traits on indicators of sperm production. (DOC) [file pone.0076510.s005.doc]

**Table S4**. Effects of spermatogenic traits on indicators of sperm production

| Dependent variable | Predictor | Slope | *F* | *P* value | *λ* | *r* | CI | n |
| --- | --- | --- | --- | --- | --- | --- | --- | --- |
| DSP | body mass | 0.02 | 13.31 | **0.001** | 0.85^*, n.s^. | 0.55 | **0.26 to 0.97** | 34 |
|  | PST | 1.27 | 18.04 | **0.0002** |  | 0.61 | **0.35 to 1.06** |  |
| SpCauda | body mass | 0.89 | 68.47 | **<0.0001** | <0.01^n.s., n.s^. | 0.86 | **0.90 to 1.70** | 27 |
|  | PST | 4.57 | 11.32 | **0.003** |  | 0.57 | **0.24 to 1.04** |  |
| SpEjac | body mass | 0.83 | 1.95 | 0.17 | 0.94^*, n.s^. | 0.25 | -0.11 to 0.62 | 32 |
|  | PST | 5.10 | 30.7 | **<0.0001** |  | 0.72 | **0.54 to 1.27** |  |
| DSP | body mass | -0.16 | 12.76 | **0.001** | 0.98^*, n.s^. | 0.57 | **0.27 to 1.04** | 29 |
|  | number of Sertoli cells | 0.37 | 2.66 | 0.12 |  | 0.30 | -0.07 to 0.70 |  |
| SpCauda | body mass | 0.58 | 17.38 | **0.002** | <0.01^n.s., *^ | 0.80 | **0.47 to 1.71** | 13 |
|  | number of Sertoli cells | -1.70 | 2.79 | 0.13 |  | 0.47 | -0.11 to 1.13 |  |
| SpEjac | body mass | 0.74 | 17.45 | **0.0005** | <0.01^n.s., n.s^. | 0.68 | **0.40 to 1.27** | 23 |
|  | number of Sertoli cells | -0.81 | 0.46 | 0.50 |  | 0.15 | -0.29 to 0.59 |  |
| DSP | body mass | -0.11 | 23.83 | **<0.0001** | 0.71^n.s., n.s^. | 0.70 | **0.47 to 1.26** | 28 |
|  | ESC | 0.97 | 27.34 | **<0.0001** |  | 0.72 | **0.52 to 1.31** |  |
| SpCauda | body mass | 0.60 | 42.15 | **0.0002** | <0.01^n.s., *^ | 0.92 | **0.88 to 2.26** | 11 |
|  | ESC | 3.05 | 29.29 | **0.0006** |  | 0.89 | **0.71 to 2.10** |  |
| SpEjac | body mass | 0.57 | 8.65 | **0.009** | 0.66^n.s., n.s^. | 0.57 | **0.19 to 1.11** | 21 |
|  | ESC | 0.57 | 0.34 | 0.57 |  | 0.14 | -0.33 to 0.60 |  |
| DSP | body mass | -0.06 | 18.72 | **0.0001** | <0.01^n.s., *^ | 0.60 | **0.35 to 1.04** | 36 |
|  | SECL | -2.1 | 35.19 | **<0.0001** |  | 0.72 | **0.56 to 1.25** |  |
| SpCauda | body mass | 0.85 | 90.1 | **<0.0001** | <0.01^n.s., *^ | 0.91 | **1.07 to 1.97** | 22 |
|  | SECL | -5.38 | 14.14 | **0.001** |  | 0.65 | **0.33 to 1.23** |  |
| SpEjac | body mass | 0.81 | 55.60 | **<0.0001** | 0.26^n.s., n.s^. | 0.79 | **0.74 to 1.42** | 36 |
|  | SECL | -3.48 | 6.13 | **0.019** |  | 0.40 | **0.08 to 0.76** |  |
| DSP | body mass | -0.06 | 2.14 | 0.16 | <0.01^n.s., n.s^. | 0.35 | -0.14 to 0.88 | 18 |
|  | spermiogenesis | -1.61 | 11.38 | **0.004** |  | 0.66 | **0.28 to 1.29** |  |
| SpCauda | body mass | 0.63 | 22.95 | **0.0004** | <0.01^n.s., *^ | 0.81 | **0.56 to 1.69** | 15 |
|  | spermiogenesis | -4.11 | 4.41 | 0.058 |  | 0.52 | **0.01 to 1.14** |  |
| SpEjac | body mass | 0.40 | 6.10 | **0.025** | 1^n.s., n.s^. | 0.53 | **0.09 to 1.07** | 19 |
|  | spermiogenesis | 1.22 | 0.73 | 0.41 |  | 0.21 | -0.28 to 0.70 |  |
| SpCauda | DSP | 1.82 | 5.21 | **0.039** | 0.75^*, n.s^. | 0.52 | **0.03 to 1.12** | 16 |
| SpEjac | SpCauda | 1.00 | 140.2 | **<0.0001** | <0.01^n.s., n.s^. | 0.95 | **1.33 to 2.41** | 16 |

Phylogenetically controlled multiple regression analyses revealing the effect of spermatogenic traits on daily sperm production, the number of sperm in the caudae epididymides, and the number of sperm in the ejaculate. All variables were log_10_-transformed (with the exception of the proportion of seminiferous tubules, which was arcsine-transformed) prior to analysis. The superscripts following the λ value indicate significance levels (n.s., p > 0.05; *, p < 0.05) in likelihood ratio tests against models with *λ* = 0 (first superscript) and *λ* = 1 (second superscript). The effect size *r* was calculated from the *F* values; we also present the non-central 95% confidence interval (CI), an interval excluding 0 indicating statistically significant relationships. The *P* values and CI that indicate statistical significance are shown in bold. Abbreviations: DSP: daily sperm production; n: number of species in each analysis; PST: percentage of seminiferous tubules; SpCauda: number of sperm in the cauda epididymides; SpEjac: number of sperm in the ejaculate; HSE: height of the seminiferous epithelium; ESC: efficiency of Sertoli cells (number of round spermatids / Sertoli cell); SECL: seminiferous epithelium cycle length.
